# Portable Multispectral Colorimeter for Metallic Ion Detection and Classification

**DOI:** 10.3390/s17081730

**Published:** 2017-07-28

**Authors:** Mauro S. Braga, Ruth F. V. V. Jaimes, Walter Borysow, Osmar F. Gomes, Walter J. Salcedo

**Affiliations:** 1 Laboratório de Microeletrônica, Escola Politécnica da Universidade de São Paulo, São Paulo 05508-010, Brazil; msbraga@lme.usp.br; 2 Instituto Federal de Educação, Ciência e Tecnologia de São Paulo, Cubatão 11533-160, Brazil; wborysow@ifsp.edu.br; 3 Centro de Ciências Naturais e Humanas, Universidade Federal do ABC, Santo Andre 09210-580, Brazil; rfvillam@iq.usp.br; 4 Centro de Capacitação e Pesquisa em Meio Ambiente (Cepema-USP), Cubatão 11540-990, Brazil; ofgomes@usp.br

**Keywords:** portable environmental monitoring systems, metallic ions detection, colorimetric system

## Abstract

This work deals with a portable device system applied to detect and classify different metallic ions as proposed and developed, aiming its application for hydrological monitoring systems such as rivers, lakes and groundwater. Considering the system features, a portable colorimetric system was developed by using a multispectral optoelectronic sensor. All the technology of quantification and classification of metallic ions using optoelectronic multispectral sensors was fully integrated in the embedded hardware FPGA ( Field Programmable Gate Array) technology and software based on virtual instrumentation (NI LabView^®^). The system draws on an indicative colorimeter by using the chromogen reagent of 1-(2-pyridylazo)-2-naphthol (PAN). The results obtained with the signal processing and pattern analysis using the method of the linear discriminant analysis, allows excellent results during detection and classification of Pb(II), Cd(II), Zn(II), Cu(II), Fe(III) and Ni(II) ions, with almost the same level of performance as for those obtained from the Ultravioled and visible (UV-VIS) spectrophotometers of high spectral resolution.

## 1. Introduction

Heavy metal ions have presented strong threats to human health as they have a lot of toxic bio-cumulative properties in the natural environment. Once these ions are thrown into rivers and lakes near cities, they can affect the vegetables and animals, unbalancing the whole food chain [1]. The main health problem caused by heavy metal ions and the threshold level in drinking water according to the World Health Organization (WHO) are summarized in Table 1. To this end, great effort has been made by the scientific community in order to develop devices and systems for metal ions detection. Electrochemical devices were initially offered, which have achieved high accuracy and, in some special cases, also high selectivity by using nanomaterials such as active electrodes [2,3,4]. However, these types of devices normally suffer interference from electromagnetic noise sources. In order to avoid this, many dye molecules or also bio-indicator molecules have been successfully used for signal detection of the absorption (colorimetric) and fluorescent emission spectra. For example, Anabas testudines were applied as a bio-indicator for Hg and Pb metal ions detection using an ion exchange chromatography spectrometer. The experimental setup of this assay needs complex procedures for the separation and purification of samples [5]. Aminopyridine shift base molecules were also used for Ni(II), Zn(II), Fe(III) and UO_2_(II) ions detection by colorimetric and flourogenic methods as a conventional spectrometer. 

All the previous procedures have showed that the colorimetric method allows selectivity for Ni(II) and Zn(II) ions, the selectivity was only for Zn(II) when applying the fluorescence technique [6]. A sucessful review paper showed that the colorimetric technique is a good suitable procedure for metal ions detection, especially when functionalized gold is used in a nanoparticle absorption spectra shift for ions detection [7]. The dyad biodipy–rhodamine molecule was utilized for three-valent ions (Al(III), Cr(III), Fe(III)) detection by monitoring changes in fluorescence emission due to energy transfer from biodipy to rhodamine moiety, this dyad molecule did not show any selectivity between these ions, as was reported. The dyad could also be manipulated as an imaging indicator in the biological cell culture [8]. A carbon dot ending with carboxylate groups was applied as a chemo sensor for the detection of many metallic ions, by using the photoluminescence quenching of these dots. However, only selectivity to Fe(III), Pb(II) and Hg(II) was achieved after buffer solution switching for each kind of ion [9]. A single pyridine-linked anthracene-based molecule was taken for the detection of various metallic ions; the change of the photoluminescence emission and its dye also presented selectivity to Pd(II) ion when an Sodium Dodecyl Sulfate (SDS) surfactant was additionally used in the sample solution [10]. A portable microfluidic system for a microbial biosensor was reported to detect Pb(II) and Cd(II) ions, for this purpose the authors used an inverted fluorescence microscopy spectrometer [11]. The Plasmon resonance fiber-optic-based sensor was chosen for metal ions detection, using the peak resonance shift [12]. A review paper reported many different carbon nanoparticle structures to detect Hg(II), Cu(II) and Fe(III) ions by fluorescent off, fluorescence on and ratiometric detection mechanisms. Even though these structures were shown to be a good potential material for metallic ion detection, they could not be precise in the selection and detection of all the mechanisms described here, as they were very sensible to buffer usage in the metallic ions solution [13]. The benzothiazolium-derived molecules were proposed as a colorimetric and fluorescent chemosensor to detect Hg(II) ions; these molecules showed high selectivity for Hg(II) ions and the colorimetric and fluorescent calibration curves were achieved by monitoring the peak position changes (i.e., a specific spectral point) of the absorption spectra and fluorescent spectra respectively [14]. The main challenge in metallic ion detection is to develop a recoverable system, it was reported for a photonic colorimetric device based in the Bragg diffraction process. In this work, the sensor was doped with hydroxyquilonine molecules and the sensor had a selective response for Pb(II) and Cu(II) ions with good reversibility [15]. The selectivity of optical sensors for metallic ion detection is still an issue to solve. Some authors reported the matrix array indicators to overcome this problem, the array of 12 different thiophene-based compounds were used to detect and classify various metallic ions, the authors suggest that 100% classification was possible when they used the fluorescence signal from the phiophene-based molecules and these signals were processed by linear discriminant analyses [16].

As we described above, all the systems that used colorimetric or fluorescence techniques used the conventional test bench spectrophotometer and the selectivity of these systems was specific for some type of metallic ions. In these contexts, this article presents the development of a portable colorimetric and fluorescent chemical detection system, for the detection of metallic ions in liquid media. The system is based on the optoelectronic multispectral sensor as the detector and the white light emitting diode has been used as an excitation source. All components of the system such as excitation, detection and test calibration curves, have been controlled by a real-time embedded national board acquisition system programed with LabVIEW software from the National Instrument Company. The system was tested using the 1-(2-pyridylazo)-2-naphthol (PAN) molecules as the colorimetric indicator and the achieved results showed that this system could detect and classify many metallic ions at the same time (Pb(II), Cd(II), Zn(II), Cu(II), Fe(III) and Ni(II). The portable system proposed, together with signal processing technique, could apply to metallic ion detection in situ environments such as rivers and lakes.

## 2. Experimental Procedures

**Figure 1:**
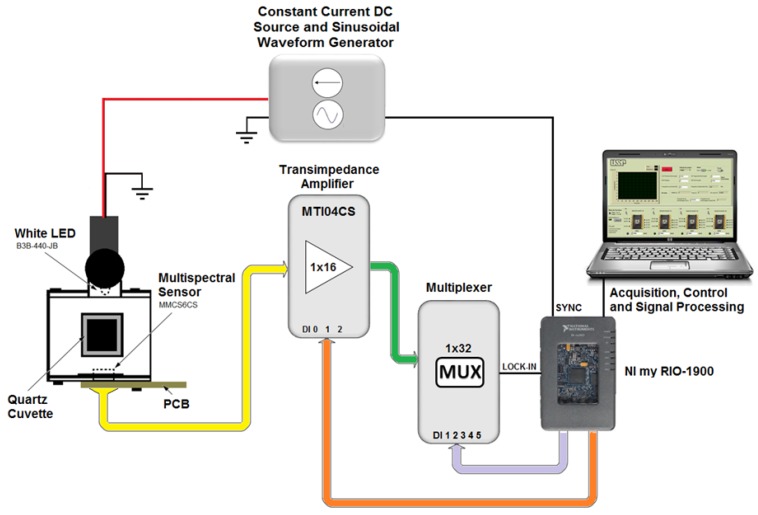
Schematic diagram of the portable colorimetric system built with a multispectral optoelectronic sensor.

The portable embedded system for the detection of different metallic ions by the colorimetric method used a photodetector optoelectronic chip, composed of 18 sets of photodiodes (3 × 6) encapsulated in a same enclosure, MMCS6CS type, manufactured by the MAZeT company (Jena, Germany). In this device, there were three groups of six photodiodes symmetrically distributed in a circular structure of 2 mm diameter. Each group of photodiodes had a spectral dielectric filter that selects the specific wavelength band so that the complete array of photodiodes covers the spectral region from 380 nm to 780 nm, where each group with specific filters is sensible to the band centered at 425, 475, 525, 625, 575 and 675 nm respectively. Additionally, there was one group of six photodiodes that did not have any filter, i.e., unfiltered array (PW). The photodiodes were connected directly to two integrated transimpedance amplifiers of MTI04CS type, which have four channels with programmable gains. The amplifiers chips were manufactured by MAZeT company (Jena, Germany). The gain selection was achieved by combining the three-bit binary entrance of the MTI04CS integrated circuit, allowing up to eight different stages of amplification levels. After the amplification step, the signals from the photodiodes are multiplexed and directed to a processing and signal acquisition module in order to get the electrical signal (V_dc_) that corresponds to a light intensity that arrived at each groups of photodiode array of the multispectral sensor. As light source, a white light-emitting diode (LED) (P_max_ = 120 mW, IF = 30 mA), manufactured by the company Laser Roithner Technik (B3B-440-JB) was used. This source was set up at the front side of the quartz cuvette that contains the sample solution. The LED was fed with constant current source. The acquisition, control and processing of the signals was performed based on Field Programmable Gate Array (FPGA) technology, which was developed based on virtual instrumentation software (NI LabView^®^), manufactured by National Instruments, NI model myRIO-1900 (Austin, TX, USA). Figure 1 shows a schematic diagram of the portable embedded system for the detection of the heavy metal ions (Cu(II), Zn(II), Ni(II), Cd(II), Pb(II), and Fe(III)) by the colorimetric method using a chromogen reagent and multispectral optical sensor. The system manufactured in this way is a portable system that can easily be plugged and played to a computer. The physical picture of the system is depicted in Figure A7 of the Appendix A.

The solution with different metal ions was prepared with reagents of 99.9% purity. All reagents were acquired from the Sigma-Aldrich Chemistry (São Paulo, Brazil) and deionized (DI) water was purified with a Milli-Q system Gradient. Standard solutions for the different metals were prepared in water DI, with a suitable dilution of 250 ppm of salts of copper sulphate (CuSO_4_), zinc sulfate (ZnSO_4_), nickel chloride (NiCl_2_), cadmium chloride (CdCl_2_), lead nitrate (Pb(NO_3_)_2_), iron(III) nitrate (FeN_3_O_9_). The pH values of the ionic solutions were read by a pH meter, LUCA-210 model, manufacturer Lucadema and unmodified according to those values obtained after the process of the dilution of salts in the water, as is shown in Table 2.

The chromogen reagent of 1-(2-pyridylazo)-2-naphthol (PAN) was diluted with methanol in order to get a concentration of 100 μM. Before each data acquisition, a volume of 2.5 mL of the prepared PAN solution was added into a quartz cuvette and then small additions of appropriate volumes of the metal ions Cu(II), Zn(II), Ni(II), Cd(II), Pb(II), Fe(III) were performed in order to get concentrations of 1 to 10 ppm, respectively. It is important to point out that all ion concentrations were authenticated by the EPA SW-846 Test Method 6010D: Inductively Coupled Plasma-Optical Emission Spectrometry using a standard of Pb-CGPB1-1 (1000 μg/mL) in 0.5% HNO_3_ (v/v) Inorganic Ventures—CAS No.: 7439-92-1, Cd-CGCD1-1 (1000 μg/mL) in 2.0% HNO_3_ (v/v) Inorganic Ventures—CAS No.: 7440-43-9, Zn-CGZN1-1 (1000 μg/mL) in 2.0% HNO_3_ (v/v) Inorganic Ventures—CAS No.: 7440-66-6, Cu-CGCU1-1 (1000 μg/mL) in 2.0% HNO_3_ (v/v) Inorganic Ventures—CAS No.: 7440-50-8, Ni-CGNI1-1 (1000 μg/mL) in 2.0% HNO_3_ (v/v) Inorganic Ventures—CAS No.: 7440-02-0, Fe-CGFE1-1 (1000 μg/mL) in 2.0% HNO_3_ (v/v) Inorganic Ventures—CAS No.: 7439-89-6.

The response of the multispectral sensor MMCS6CS in the presence of metal ions of Pb(II), Cd(II), Zn(II), Cu(II), Fe(III) and Ni(II) was based on spectral change measurement of the optical transmittance spectra of a PAN solution, due to the action of different ions. In this case, the light intensity transmitted and received by the array of photodiodes was converted by transimpedance amplifiers (MTI04CS) into V_DC_ voltage values and stored by the acquisition, control and processing module (myRIO-1900). The transmittance was determined relative to the reference signal which corresponded to the response of each photodiode in different arrays to the transmitted white light through the solvent used in the preparation of the solution samples.

Before each signal reading, a volume of 2.5 mL of prepared PAN solution (100 μM) was added into a cuvette of quartz which has square shape of 10 mm each side. After this, a calibrated pipette, model P100 (20–100 μL), Gilson Pipetman, was used to add a small volume of metal ions diluted in water in order to get a concentration of ions in the range of 1 to 10 ppm. During the experiment, the ambient temperature was kept at 26 °C.

## 3. Results and Discussion

**Figure A1:**
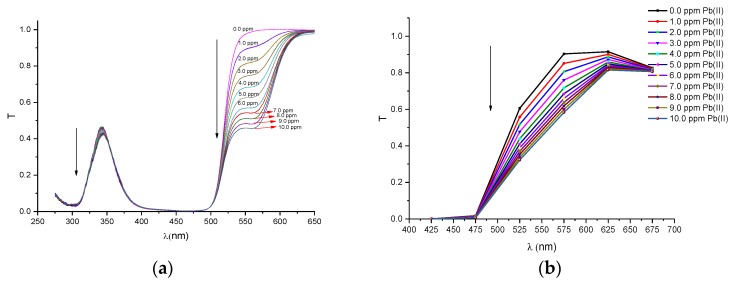
The transmittance spectra of the chromogen reagent of 1-(2-pyridylazo)-2-naphthol (PAN) solution containing Pb(II) ions at different concentrations corresponding to: (**a**) The UV-VIS Cary^®^ 50-Varian spectrometer (**b**) the multiespectral MMCS6CS sensor.

**Figure A2:**
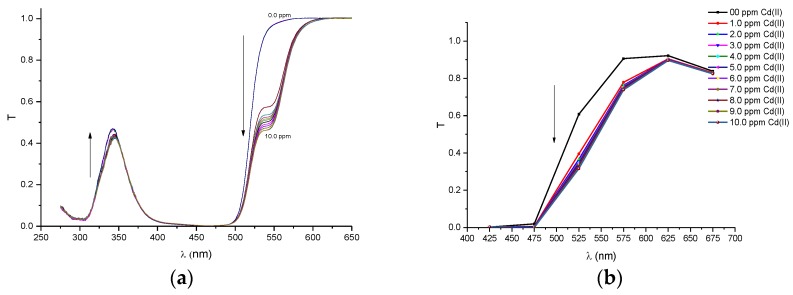
The transmittance spectra of the chromogen reagent of 1-(2-pyridylazo)-2-naphthol (PAN) solution containing Cd(II) ions at different concentrations corresponding to: (**a**) The UV-VIS Cary^®^ 50-Varian spectrometer (**b**) the multiespectral MMCS6CS sensor.

**Figure A3:**
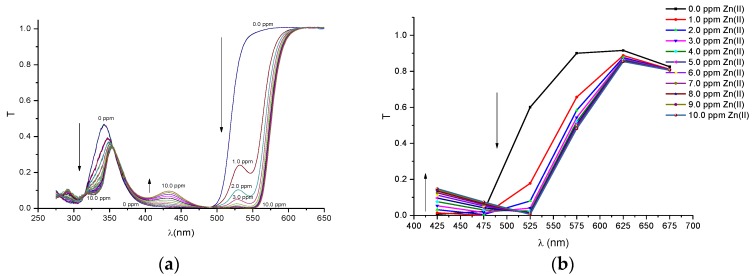
The transmittance spectra of the chromogen reagent of 1-(2-pyridylazo)-2-naphthol (PAN) solution containing Zn(II) ions at different concentrations corresponding to: (**a**) The UV-VIS Cary^®^ 50-Varian spectrometer (**b**) the multiespectral MMCS6CS sensor.

**Figure A4:**
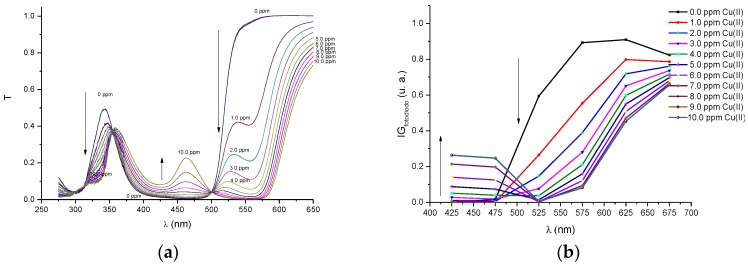
The transmittance spectra of the chromogen reagent of 1-(2-pyridylazo)-2-naphthol (PAN) solution containing Cu(II) ions at different concentrations corresponding to: (**a**) The UV-VIS Cary^®^ 50-Varian spectrometer (**b**) the multiespectral MMCS6CS sensor.

**Figure A5:**
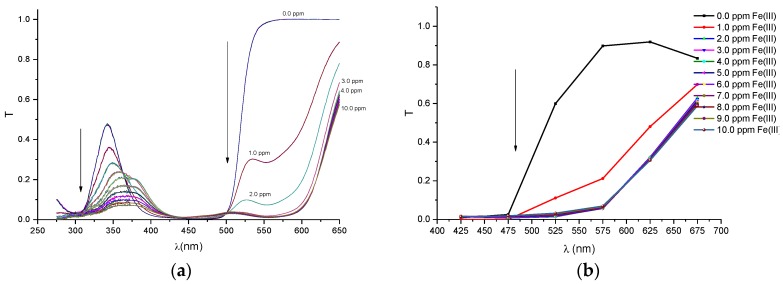
The transmittance spectra of the chromogen reagent of 1-(2-pyridylazo)-2-naphthol (PAN) solution containing Fe(III) ions at different concentrations corresponding to: (**a**) The UV-VIS Cary^®^ 50-Varian spectrometer (**b**) the multiespectral MMCS6CS sensor.

**Figure A6:**
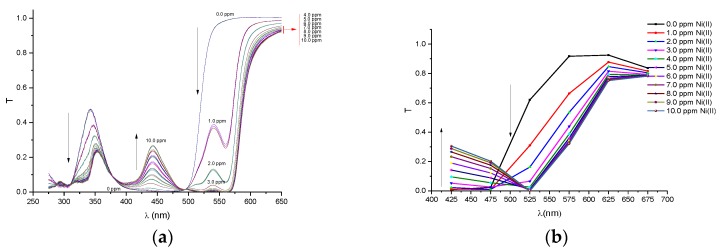
The transmittance spectra of the chromogen reagent of 1-(2-pyridylazo)-2-naphthol (PAN) solution containing Ni(II) ions at different concentrations corresponding to: (**a**) The UV-VIS Cary^®^ 50-Varian spectrometer (**b**) the multiespectral MMCS6CS sensor.

**Figure A7:**
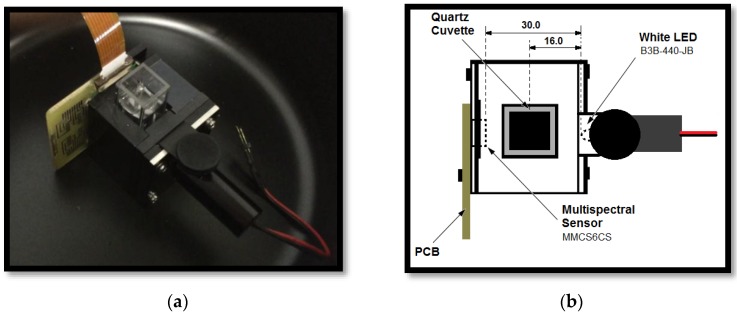
(**a**) Picture of the portable system with the detection sensor MMCS6CS set at the front of the source of a white light-emitting diode (LED). (**b**) Schematic representation of the portable system.

In order to compare the performance of our proposed portable colorimeter first, the transmittance spectra (T) was obtained of PAN (sensitive molecule) and of the solutions of this molecule in environments containing metal ions of Pb(II), Cd(II), Zn(II), Cu(II), Fe(III) and Ni(II), respectively. The spectra were obtained by a UV-VIS spectrometer Cary 50 model, Varian and are presented in Figure A1a, Figure A2a, Figure A3a, Figure A4a, Figure A5a and Figure A6a, which can be seen in the supplementary information (Appendix).

Then, the portable colorimeter which was built with the multispectral sensor MMCS6CS was used to obtain the transmittance spectra of the PAN solution containing the different metallic ions so that the solutions had the same condition as the ones used with the UV-VIS spectrometer. The transmitted light signals were detected with the six photodetector output terminals (MAZeT) that correspond to the responses of the array of photodiodes with band pass optical filters centered at 425, 475, 525, 575, 625 and 675 nm, respectively. These signals were conditioned using the digital LOCK-IN amplification process. The transmittance spectra were determined comparing the signal from the PAN solution relative to the signal corresponding to the solvent (methanol) used for the PAN solution preparation. Equation (1) gives the transmittance relation that was obtained using the detected signals on the photodiode array.

(1) $$T=\frac{I_{\mathrm{sample}\;(\lambda )}}{I_{\mathrm{solvent}\;(\lambda )}}$$

where *I*_sample_ (*λ*) is the current generated by the photodiode array with an optical filter centered at the wavelength *λ* when the samples were the PAN solution without or with metal ions, respectively. *I*_solvent_ is the current generated by the photodiode array with an optical filter centered at the wavelength *λ* when the sample only corresponds to a solvent (methanol).

The transmittance spectra for different metal ions and at different concentrations obtained this way are depicted in the Figure A1b, Figure A2b, Figure A3b, Figure A4b, Figure A5b and Figure A6b in the supplementary information (Appendix A).

The spectra results with the UV-VIS spectrometer and portable system clearly show that the presence of metal ions in the solutions of the PAN molecules changes the profile of the transmittance bands and these changes are related to the change in color of the original solution (PAN solution free of ions). The color change mechanism could be explained as follows: the PAN molecule is composed of two aromatic groups, the pyridyl group and naphthol group, joined by azonitrogen. The aromatic groups act as an optical antenna in the UV-VIS region. The interaction of the PAN molecule and the metallic ion in a solution promoted a reaction such that the PAN acts as a tridentate ligand complexing with metal ions through the ortho-hydroxyl group of naphthol rings and the azonitrogen approach hetrocyclic nitrogen atom. This reaction promotes changes in the electronic orbital of pyridyl and naphthol groups which are responsible for the absorption spectrum of the PAN molecule in the UV-VIS region. Thus, the PAN molecule chelation with metal ions changes its spectral band absorption shape and these band changes are used as indicators to identify different types of metallic ions [18,19].

The spectra, obtained with the multispectral sensor MMCS6CS, certainly have lower quality than the spectra obtained with the conventional UV-VIS spectrometer, since the multispectral sensor system has a discrete number of spectral points (six filtered sensors). However, it can be observed in the Figure A1b, Figure A2b, Figure A3b, Figure A4b, Figure A5b and Figure A6b that the profiles of the discrete spectra follow the same trend in the change of spectra that were obtained with the UV-VIS spectrophotometer. It is important to point out that the spectral range of the set of six filtered sensors was limited to a range between 380 and 780 nm. In this sense, in order to obtain a more accurate comparison, the region of the wavelength bands in the ultraviolet region (275–375 nm), seen in the spectra with the UV-VIS spectrometer, were suppressed for the quantitative analyses.

Before the colorimetric analyses, the sensitivity response of our proposed system was compared with that obtained with conventional spectrometers. For this proposal, the transmittance coefficient was analyzed at 525 nm, which is a sensible spectral point that changes significantly with metal ion concentrations. Thus, we define a response function at this point to both the spectrometer and the multispectral MMCS6CS system, using the following Equation (2).

(2) $$\mathrm{Response}=\frac{T_{0}-T}{T_{0}}$$

where *T*_0_ and *T* are the transmittance coefficients of the PAN solution without and with metallic ions, respectively.

**Figure 2:**
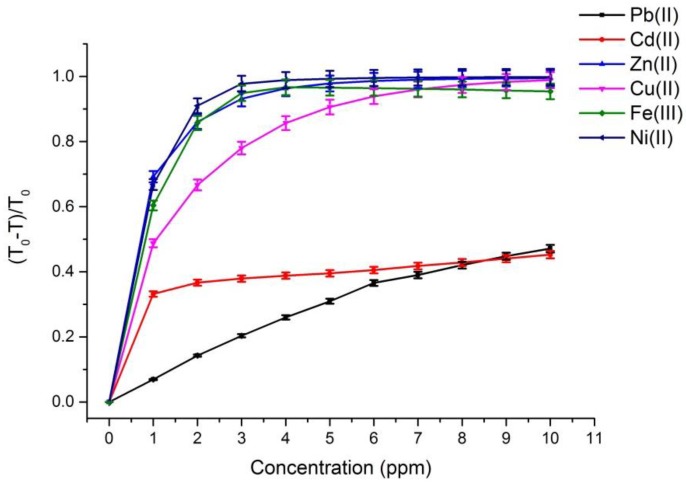
The response curves obtained from the transmittance spectra at 525 nm which were achieved with the Ultraviolet-visible (UV-VIS) stectrophotometer for different metallic ions at different concentrations. The measurements were repeated ten times and the fluctuations of each experimental point were about 0.01%. The error bars were calculated considering the transmittance error of the spectrometer 2.5%.

**Figure 3:**
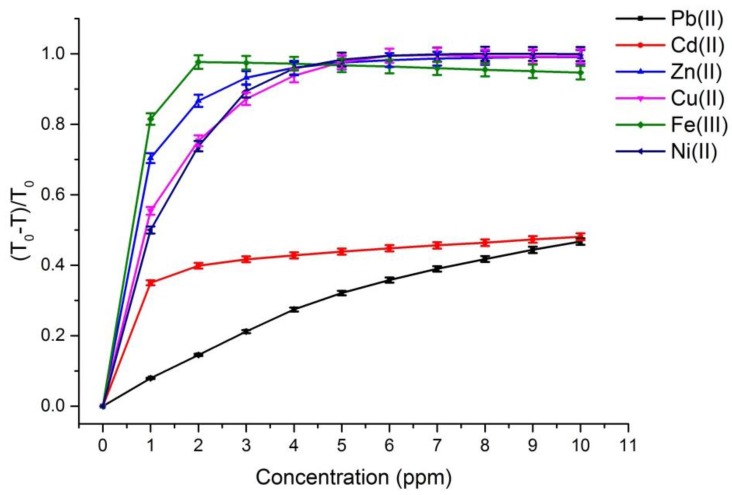
The response curves obtained from the transmittance spectra at 525 nm which were achieved with a portable colorimetric system based in a multispectral sensor for different metallic ions at different concentrations. The measurements were repeated ten times and the fluctuations of each experimental point were about 0.1%. The error bars were calculated considering the error in the photocurrent measurement of the multispectral sensor 1.7% (in the worst case).

Figure 2 and Figure 3 depict the calibration curves of the responses obtained by UV-VIS spectrometer and multispectral sensor MMCS6CS, in different concentrations of metals ions, at a wavelength of 525 nm, respectively. It is observed that, for both systems, the response curves for this spectral point (525 nm) present the same profile, showing the compatibility of the sensitive results of our proposed system with the results obtained by a conventional UV-VIS spectrometer.

On the other hand, Figure 2 and Figure 3 show that the response curves saturate early, showing the high sensitivity of PAN molecules to detecting the metal ions studied in this work, except for Pb and Cd ions. Considering that the response error in our proposed system (MMCS6CS) was about 1.7% (in the worst case), the limit of detection of our system was estimated by using the slope of the linear part of the response curves (Figure 3) in Equation (3) [20]. These limits of detection for all the ions studied in this work are showed in the Table 3.

(3) $$DL=\frac{3.3\sigma }{S},$$

where: *S* is the slope of response curves (linear region) and *σ* is the imprecision of the detection system (error).

The limit of detection for the Pb(II) ion is really close to the limit level for drinking water (Table 1). However, the limit of detection of the Cd(II) ion is greater in one order of magnitude than to the limit level for drinking water. The limit of detection for the Fe(III), Z(II), Cu(II) and Ni(II) ions are much smaller than the limit levels of these ions in drinking water (Table 1). These results showed that the multispectral portable system proposed in this work could be used successfully to control the water quality.

The most relevant results reported in this work are related to the classification power of different metallic ions achieved with the proposed portable colorimeter system. The classification procedure was achieved by using the Fisher linear discriminant analysis. For this procedure we used a set of 20 data for each type of ion, of which ten data were used for the training process and the other ten data were used for the testing process. In the case of the spectra from the UV-VIS spectrometer, first the transmittance curves were fitted with seven harmonic functions (Equation (4)).

(4) $$T\left(\lambda \right)=A_{0}+\;\sum_{j=1}^{7}\left[A_{j}sin\left(jK\lambda \right)+B_{j}cos\left(jK\lambda \right)\right],$$

where *λ* is the wavelength of excited light; and *K* is the fundamental frequency of the harmonic series.

The sixteen parameters *K*, *A_j_* and *B_j_* (*j* = 1, 2, …, 7) were used as the input data for the linear discriminant analyses (training and testing process). It is important to point out that different authors proposed a classification process using the colorimetric technique by using the spectral point where the significant variation of the transmittance (or absorbance) coefficient happened [9]. This strategy certainly loses the profile change of all the transmittance bands. In this regard, the fitting process proposed in this work preserved the intensity and shape variation of the spectral bands on the classification process.

In the case of the multispectral MMCS6CS portable colorimeter, we have the six spectral points for the transmittance spectra, so these six transmittance coefficients were directly used as the input data for the linear discriminant analyses.

**Figure 4:**
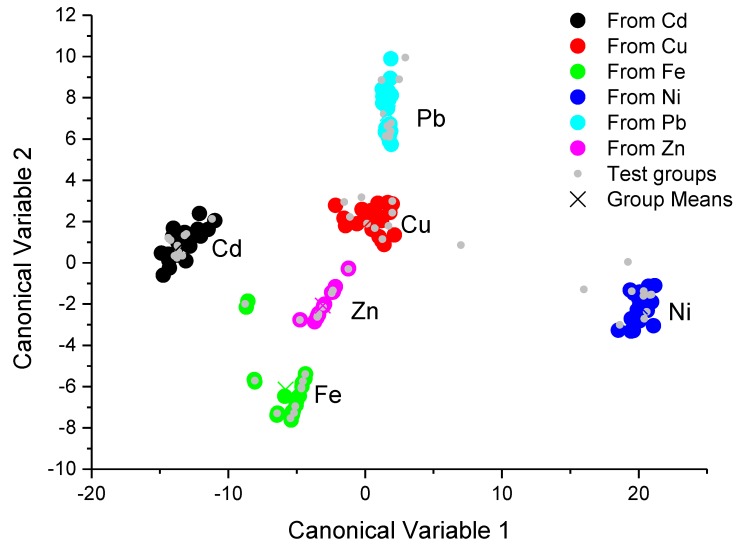
The canonical score plots for the training and testing processes built from the set of spectra data which were obtained with the Ultravioled-visible (UV-VIS) bench spectrometer. The classification procedure was obtained by using the linear discriminant analyses.

**Figure 5:**
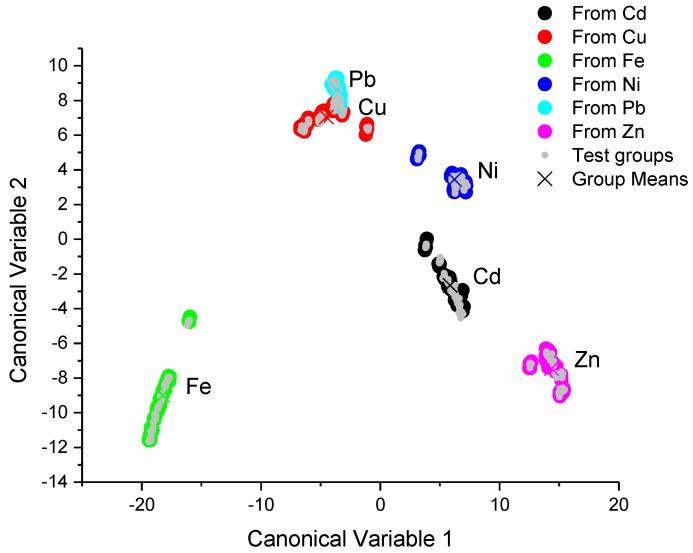
The canonical score plots for the training and testing processes built from the set of spectra data which were obtained with a portable colorimetric system based in multispectral sensors. The classification procedure was obtained by using linear discriminant analyses.

The canonical score plots for the training and testing process are depicted in the Figure 4 and Figure 5, respectively.

Figure 4 shows that the training process achieved an excellent classification for the spectra data obtained with the UV-VIS spectrometer, since the different classes were clearly separated between them by hyperplanes. This figure also shows that the testing results and the error rates for all metallic ion recognition were 0%, as is shown in Table 4.

The score plot of the training and testing process, which were obtained from the proposed portable colorimeter, is depicted in Figure 5. The clusters of different classes were almost totally separated by hyperplanes, except for the clusters corresponding to Cu(II) and Pb(II) ions, where it was not possible to draw a hyperplane which could separate these clusters. The testing process also shows an error rate for Cu(II) ion recognition of 10%, as can be seen in Table 3—i.e., 10% of Cu(II) ion samples were misunderstood as Pb(II) ions. Even though it was not possible to understand the samples for Cu(II) and Pb(II) ions, all the other metallic ions used in this work were successfully classified with an error rate of 0% (Table 5). The classification results obtained with the portable multispectral colorimetric system almost showed equivalent performance with those obtained with the conventional UV-VIS spectrometer, the 10% of misunderstood Cu(II) and Pb(II) ions must be due to discrete spectral points of the multispectral detector of our system, which loses fine details of band shape changes.

## 4. Conclusions

In the present work, a portable device system applied in the detection of different metallic ions was proposed and developed, aiming at its application in the monitoring of hydrological systems like rivers, lakes and groundwater. A portable colorimetric system was designed and developed, embedded in the board acquisition of National Instruments. The system functioned as a colorimeter by using the chromogen reagent of 1-(2-pyridylazo)-2-naphthol (PAN) as an indicator, along with signal processing and pattern analysis using the linear discriminant analysis method, allowing us to obtain excellent results in the detection and classification of Pb(II), Cd(II), Zn(II), Cu(II), Fe(III) and Ni(II) ions, with almost the same level of performance as those obtained from UV-VIS spectrometers with high spectral resolution. All the technology for the quantification and classification of metallic ions using optoelectronic multispectral sensors was fully integrated into the embedded hardware FPGA technology and software based on virtual instrumentation (NI LabView^®^).

The portable system developed in this work suggests its application for environmental control in situ and in real time, in such a way that it can be integrated into a network of sensors that can provide data continuously and receive commands to control environmental monitoring centers. In addition, the proposed system can be applied for the detection of various types of gases simultaneously, since the different dye molecules sensitive to different types of gas and with different spectral responses could be integrated into the active area of multispectral sensors. In this case it will be used for the absorption or photoluminescence spectra of dye molecules since our portable system provided an easy process for switching the source of a white-light-emitting diode (used for absorption spectra obtention) by an emitting laser diode at a specific wavelength, which can be used as a source to excite the dye molecules for photoluminescence emission.

## Figures and Tables

**Table 1:** Limit of various heavy metal ions in drinking water according to World Health Organiztion (WHO).

Metal Ions	WHO Limit mg/L (ppm) [17]	Effects [1]
Cu(II)	2	Alliergies, anaemia, kidney disorder
Zn(II)	3	Respiratore disorder, neuronal disorder, prostate cancer
Ni(II)	0.07	At hig level may be toxic, even carcinogenic
Cd(II)	0.003	Renal toxicity, hypertension, lymphocytosis, pulmonary fibrosis, lung cancer, osteoporosis, hyperuricemia
Pb(II)	0.05	Penetrates through protective blood brain barrier, Alzheimer’s disease and senile dementia, neuro degenerative diseases, kigney damage
Fe(III)	3	At high level may be originated hemochromatosis, damage cell in the hear liver
As(III)	0.05	Causes effect on central nervous system, cardio vascular and pulmonary diseases, anorexia, gastrointestinal disease, hyper pigmentation, skin cancer
Ag(I)	0.1	Argyria, gastroenteritis, neuronal disorder, mental fatigue, rheumatism
Cr(VI)	0.05	Reproductuve toxicity, embryotoxicity, mutagenicity, carcinogenicity, lung cancer, dermatitis, skin ulcers
Hg(II)	0.001	Impared neurologic development, effects on digestive system, immune system, hypertension

**Table 2:** The pH values of prepared ionic solutions for colorimetric assays.

Ionic Solution of 250 ppm	pH
Cu(II)	4.0
Zn(II)	4.5
Ni(II)	4.5
Cd(II)	4.5
Pb(II)	4.5
Fe(III)	3.0

**Table 3:** Detection limit (DL) of the MMCS6CS system.

Metal Ions	DL (ppm)
Fe(III)	0.0068
Zn(II)	0.0079
Cu(II)	0.010
Ni(II)	0.011
Cd(II)	0.016
Pb(II)	0.077

**Table 4:** Classification counts and error rates of different metallic ions obtained after processing and analyzing the fitted parameter of transmittance spectra (obtained with UV-VIS spectrometer) by the linear discriminant analysis method.

	**Predicted Group**						
	**Cd(II)**	**Cu(II)**	**Fe(III)**	**Ni(II)**	**Pb(II)**	**Zn(II)**	**Total**
Cd	20	0	0	0	0	0	20
	**100.00%**	0.00%	0.00%	0.00%	0.00%	0.00%	100.00%
Cu	0	20	0	0	0	0	20
	0.00%	**100.00%**	0.00%	0.00%	0.00%	0.00%	100.00%
Fe	0	0	20	0	0	0	20
	0.00%	0.00%	**100.00%**	0.00%	0.00%	0.00%	100.00%
Ni	0	0	0	20	0	0	20
	0.00%	0.00%	0.00%	**100.00%**	0.00%	0.00%	100.00%
Pb	0	0	0	0	20	0	20
	0.00%	0.00%	0.00%	0.00%	**100.00%**	0.00%	100.00%
Zn	0	0	0	0	0	20	20
	0.00%	0.00%	0.00%	0.00%	0.00%	**100.00%**	100.00%
Total	20	20	20	20	20	20	120
	**Error Rate**						
	**Cd(II)**	**Cu(II)**	**Fe(III)**	**Ni(II)**	**Pb(II)**	**Zn(II)**	**Total**
Prior	0.16667	0.16667	0.16667	0.16667	0.16667	0.16667	
Rate	0.00%	0.00%	0.00%	0.00%	0.00%	0.00%	0.00%

**Table 5:** Classification counts and error rates of different metallic ions obtained after processing and analyzing the output signal from the multispectral sensor by the linear discriminant analysis method.

	**Predicted Group**						
	**Cd(II)**	**Cu(II)**	**Fe(III)**	**Ni(II)**	**Pb(II)**	**Zn(II)**	**Total**
Cd	50	0	0	0	0	0	50
	**100.00%**	0.00%	0.00%	0.00%	0.00%	0.00%	100.00%
Cu	0	45	0	0	5	0	50
	0.00%	**90.00%**	0.00%	0.00%	10.00%	0.00%	100.00%
Fe	0	0	50	0	0	0	50
	0.00%	0.00%	**100.00%**	0.00%	0.00%	0.00%	100.00%
Ni	0	0	0	50	0	0	50
	0.00%	0.00%	0.00%	**100.00%**	0.00%	0.00%	100.00%
Pb	0	0	0	0	50	0	50
	0.00%	0.00%	0.00%	0.00%	**100.00%**	0.00%	100.00%
Zn	0	0	0	0	0	50	50
	0.00%	0.00%	0.00%	0.00%	0.00%	**100.00%**	100.00%
Total	50	45	50	50	55	50	300
	**Error Rate**						
	**Cd(II)**	**Cu(II)**	**Fe(III)**	**Ni(II)**	**Pb(II)**	**Zn(II)**	**Total**
Prior	0.16667	0.16667	0.16667	0.16667	0.16667	0.16667	
Rate	0.00%	10.00%	0.00%	0.00%	0.00%	0.00%	1.67%
